# Investigation of Class I Integron in *Salmonella infantis* and Its Association With Drug Resistance

**DOI:** 10.5812/jjm.10019

**Published:** 2014-05-01

**Authors:** Fariba Asgharpour, Ramazan Rajabnia, Elaheh Ferdosi Shahandashti, Mahmood Amin Marashi, Mahya Khalilian, Zahra Moulana

**Affiliations:** 1 Department of Microbiology,paramedical Sciences,Babol University of Medical Sciences,Babol, IR Iran; 2 Infectious Diseases Research Center, Babol University of Medical Sciences, Babol, IR Iran; 3 Department of Microbiology & Immunology, Faculty of Medicine, Babol University of Medical Sciences, Babol, IR Iran; 4 Department of Microbiology and Immunology, Alborz University of Medical Sciences, Karaj, IR Iran; 5 Department of Pathobiology, Tehran University of Medical Sciences, Tehran, IR Iran

**Keywords:** *Salmonella*, Class 1 Integron, Antibiotic Resistant, *Salmonella Infantis*

## Abstract

**Background:**

Infection with non-typhoid *Salmonella* (NTS) is one of the most important health problems all over the world. Antimicrobial drug resistance is increasing among *Salmonella infantis *species.

**Objectives:**

The aim of this study was to investigate the frequency of presence of class 1 integrons in *S. infantis* species as well as its association with drug resistance.

**Materials and Methods:**

This cross-sectional study was performed on 50 *S. infantis* isolated strains, collected from chicken samples between 2009-2011. These strains were identified by standard biochemical tests and serology. Antibiotic susceptibility profiles and minimum inhibitory concentration determination for 14 antibacterial agents were performed using micro dilution and disk diffusion methods. The detection of class 1 integron was performed by the PCR method. The demographic and microbiological data for the integron positive and negative isolates were compared by SPSS software.

**Results:**

Eighteen out of 50 (36%) of isolated *S. infantis *species had *intl* gene. The isolated bacteria were sensitive to cefotaxime and ciprofloxacin (100%). Also isolates were resistant to nalidixic acid, tetracycline and streptomycin. All isolate with class 1 integron were multidrug resistant.

**Conclusions:**

The result of this study showed that due to increased level of drug resistance in *S. infantis* and the presence of class 1 integron in these strains, resistance can be transferred to other food borne pathogens.

## 1. Background

Infection with non-typhoid *Salmonella* (NTS) spp is one of the most important health problems all over the world (1). In the last two decades, antibiotic-resistant *Salmonella* species have emerged originating from food-products (2). In many countries, *Salmonella infantis* has been reported as a cause of human salmonellosis among *S. enterica* serotypes (3-6). In Europe,* S. enterica* and *S. typhimurium* have been the third most common serotypes (1.1%) followed by *S. enteric. *Furthermore, *S. infantis* strains isolated from the human and non-human resources have shown increased resistance to multiple drugs (5, 7). In many studies in Iran, the prevalence of *Salmonella* is shown to be increasing in both human and food-product samples (8, 9). Chicken is one of the main reservoirs for *Salmonella* in Iran (10). 

Antimicrobial resistance of this pathogen may lead to the spread of virulence species. Multidrug resistance (MDR) *Salmonella* has become a major problem for the medical and veterinary communities in many countries including Iran (10-12). Factors contributing to resistance and virulence may be located on chromosomes, plasmids, transposons, integrons or phages (13, 14). Integrons are DNA elements that can transfer antibiotic resistance genes among bacteria (2). Class I integrons are the most common type of integrons recognized among the multidrug-resistant *Salmonella* (15). In recent years, various studies have been implemented in industrial countries in the field of antimicrobial resistance investigating integrons in food borne *Salmonella* (14, 16-19). But, information regarding the type and distribution of antimicrobial resistance-associated integrons among food transmitted pathogens is very limited in Iran.

## 2. Objectives

The aim of the present study was to find out the occurrence and frequency of multidrug resistance species and associated integron genes in *Salmonella* isolates from chicken in Iran.

## 3. Materials and Methods

### 3.1. Bacterial Isolates

#### 3.1.1. Sampling and Isolation of Salmonella

This cross-sectional study was performed on 50 *S. infantis* isolated strains, collected from 135 chicken samples between 2009-2011. Serotyping of *Salmonella* spp, isolates was done by slide agglutination kits (Bio Merieux, France; DIFCO, USA) and compared with Kauffmann-White scheme (20). Pure colonies of each isolate of *Salmonella* were collected in two sterile 1.5 micro tubes; in a way that one of them contained one mL distilled water and the other one ml physiological serum. These samples were kept in-20°C for performing sensitivity test and DNA extraction.

#### 3.1.2. Antimicrobial Susceptibility Testing

From 28 antibiotics commonly used in veterinary and medicine, 14 antibiotics were selected including Gentamicin ((GM: 10 µg), Trimethoprim-Sulfametoxasol (SXT: 5µg), Nalidixic acid (NA: 30 µg), Ciprofloxacin (CRO: 30 µg), Cefotaxime (CTX: 30 μg), Imipenem (IPM: 10 µg), Colistin (CL: 10 µg), Ceftazidime (CAZ: 30 μg), Amoxicillin (AMX: 30 µg), Ampicillin (Amp: 10 µg), Chloramphenicol (C: 30 µg), Streptomycin (S: 10 µg), and Tetracycline (TE: 30 µg). The selected antibiotics were tested using two methods including disc diffusion (Kirby-Bauer) according to the recommendations of standard protocol of CLSI 2011 (21). 

Minimum Inhibitory Concentrations (MIC) were determined by the micro dilution method in Mueller–Hinton broth and the MIC breakpoint levels and concentration ranges of each antimicrobial agent were as follows: 512-2048 µg/mL for Nalidixic acid; 8-64 µg/mL for Tetracycline; 64-> 1024 µg/mL for Streptomycin; 8-256 µg/mL for Chloramphenicol; 512-> 2048 µg/mL for Trimethoprim and 0.01-8 µg/mL for ceftazidime, according to the recommendations of the Clinical and Laboratory Standard Institute’s (CLSI) guidelines (21). These antibiotics were purchased from Sigma Chemical Company.

#### 3.1.3. DNA Extraction

For DNA extraction, the High Pure PCR Template Preparation Kit (Roche, Germany) was used. After extracting the DNA, each sample was kept in-20°C until the PCR stage.

### 3.2. Class 1 Integron PCR

The primer sequences were as follows: R5′-CCC GAG GCA TAG ACT GTA-3′ and F5′-CAG TGG ACA TAA GCC TGT TC-3′ for the amplification of *intl* gene, which would produce a 164 base pair product. The PCR reaction was performed in final volume of 50 µL which contained 10 µL extracted DNA (equal to 1 µg), 5 pmol/L from each primer, 1.5 mmol/L MgCl_2_, 0.2 mmol/L dNTPs, and 1.5 unit of Taq enzyme (22, 23). The amplification reaction for *int1* gene included primary denaturation at 94°C for 4 minutes and then, was followed by 35 cycles of denaturation at 94°C for 30 seconds, annealing at 55°C for 30 seconds, and extending at 72°C for 30 seconds. Moreover one cycle of the final extension at 72°C for five minutes was performed (8). PCR reaction was conducted in the presence of positive and negative controls. After performing PCR reaction, electrophoresis of PCR products was conducted in 1.5% agarose gel for 60 minutes. The gel was stained with 2 µg/mL ethidium bromide. Then, the results were evaluated under UV light on the UV gel document system. After PCR, the specific band with correct size was considered as a fragment of *intl* gene.

### 3.3. Statistical Analysis

The data was analyzed using the SPSS statistical software version 18. The Chi-square test was employed to calculate the P values in terms of number of antibiotic resistant integron positive and negative isolates. The χ2 test, or the Fishers’ exact test, when appropriate, was used in a univariate analysis to assess the differences between two groups of isolates. P values less than 0.05 were considered as statistically significant.

## 4. Results

The isolated bacteria were sensitive to cefotaxime and ciprofloxacin (100%). Also, all isolates were resistant to nalidixic acid, tetracycline and streptomycin. Furthermore, twenty-three multi-drug resistance patterns were demonstrated and indicated that 16% (n = 8) and 12 %( n = 6) of these isolates were resistant to at least four and nine antimicrobial agents, respectively. The most common phenotypic patterns were related to six antibiotics (34%). Moreover, MIC_90_ and MIC_50_ values were evaluated for the six antibiotics with the highest resistance rate for all isolates. These results indicated that all isolates were resistant to nalidixic acid and trimethoprim (MIC > 2048 µg/L), 94% were resistant to tetracycline (MIC = 64 µg/L), 86% were resistant to streptomycin (MIC = 1024 µg/L), 72% were resistant to chloramphenicol (MIC = 256 µg/L) and 20% were resistant to ceftazidime (MIC = 8 µg/L) (Table 1). 

**Figure 1.**
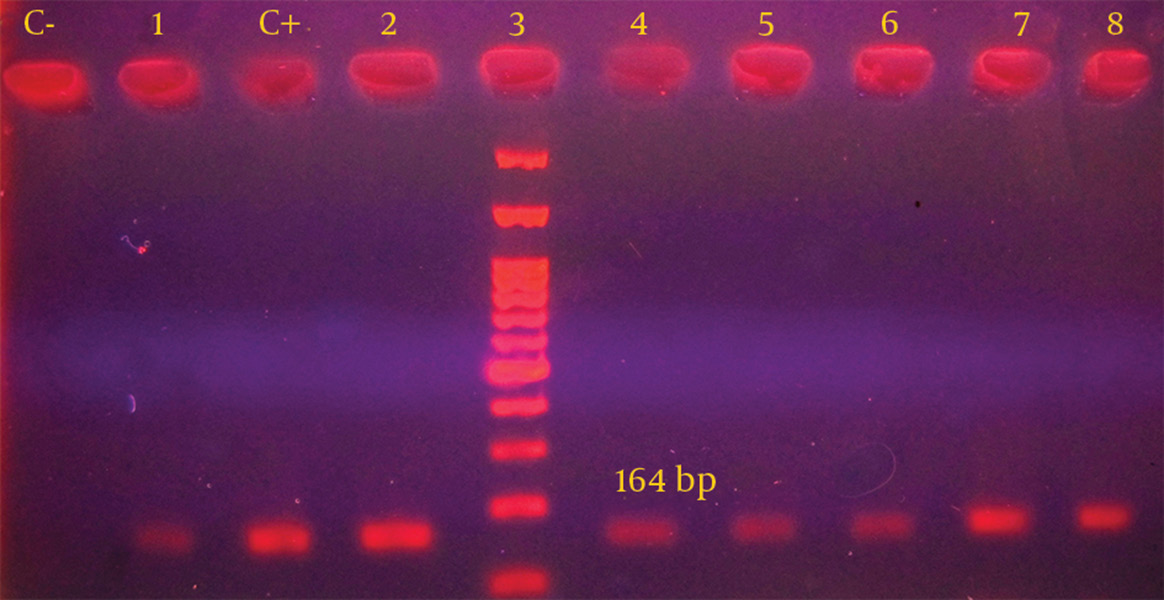
Gel-Electrophoresis of the PCR Products of the Examined* S. infantis* Strains DNA size marker=100 bp; C-, negative control; C+, positive control of *Int*-1; lane 1-3, examined strains.

Furthermore, PCR analysis was performed for all isolates and class I integron gene was detected in 18 out of 50 strains (36%) (Figure 1). Distribution of class I integron gene in association with the disc diffusion susceptibility tests are shown in Table 2. These findings demonstrated that 100% of integron-positive strains were resistant to nalidixic acid, tetracycline and streptomycin. No significant relation was e seen between the presence of integron gene and the resistance to the antibiotics. In addition, the findings obtained from the current study indicated that all isolates with multi drug resistant patterns had class I integron gene.

**Table 1.** Comparison of the Degree of Sensitivity and Resistance Rate of S.* infantis* by two Methods of Disk Diffusion and Micro Dilution Tube ^a^, ^b^

Antimicrobial Agent	MIC, µg/mL			MIC, %	DD, %	P Value
	Range	MIC_50_	MIC_90_	R (N)	R (N)	
**Nalidixic acid**	512-2048	614	2048	100 (50)	100 (50)	NS
**Tetracycline**	8-64	16	32	94 (47)	100 (50)	NS
**Streptomycin**	64- > 1024	512	1024	86 (43)	100 (50)	0.0012
**Chloramphenicol**	8-256	64	128	72 (36)	64 (32)	NS
**Trimethoprim**	512- > 2048	2048	> 2048	100 (50)	66 (33)	> 0.001
**Ceftazidime**	0.01-8	0.5	1	20 (10)	28 (14)	NS

^a^ Abbreviations: DD, Method of Disk Diffusion; MIC, Minimum Inhibitory Concentrations Method; MIC50, minimal concentration, inhibits 50% of analyzed strains; MIC90, minimal concentration, inhibits 90% of analyzed strains; NS, not significant.

^b^ Data are presented in No. (%).

**Table 2.** Frequency Distribution of Class I Integron Gene in *S. Infantis *Based on Disc Diffusion Results ^a^, ^b^

antimicrobial Agent	Disc Diffusion (n = 50)	Integron Negative (n = 32)	Integron Positive (n = 18)	P Value
**Nalidixic acid**	100 (50)	100 (32)	100 (18)	NS
**Tetracycline**	100 (50)	100 (32)	100 (18)	NS
**Streptomycin**	100 (50)	100 (32)	100 (18)	NS
**Amoxicillin**	70 (35)	78.1 (25)	55.6 (10)	NS
**Chloramphenicol**	64 (32)	65.7 (21)	61.1 (11)	NS
**Trimethoprim**	66 (33)	72 (22)	61.1 (11)	NS
**Amoxi-Clavulanic acid**	32 (16)	40.6 (13)	22.3 (3)	NS
**Ceftazidime**	28 (14)	12.5 (4)	50 (10)	0.009
**Colistin**	24 (12)	25 (8)	12.5 (4)	NS
**Ampicillin**	10 (5)	6.2 (2)	16.6 (3)	NS
**Gentamicin**	2 (1)	3.1 (1)	0 (0)	NS
**Imipenem**	0 (0)	0 (0)	0 (0)	NS
**Cefotaxime**	0 (0)	0 (0)	0 (0)	NS
**Ciprofloxacin**	0 (0)	0 (0)	0 (0)	NS

^a^ Data are presented in No. (%).

^b^ P < 0.05 was considered significant.

## 5. Discussion

Although integron class 1 plays an important role in creating and transferring the antibiotics resistance and its wide prevalence is alarming for infections caused by this bacterium (24). The results of the present study demonstrated that 18 strains of *S. infantis* (36%) contained class I integron. In other studies, the frequency of class I integron was reported to be 11-66% among different human and animal sources (16). The use of antimicrobial compounds in animal food increase the rate and spreading of antimicrobial resistance among *Salmonella* strains (25). During recent decades, different serotypes of *Salmonella* have become increasingly resistant to common antibiotics, and the appearance of multi resistance to effective antibiotics in clinic has become a health concern for health authorities in developing countries (2). 

According to several studies from Iran, the incidence of infection and multi-drug resistance to *S. infantis* is increasing in human and foods (8, 9). Drug susceptibility testing in *S. infantis* strains showed that 100% were sensitive to nalidixic acid, tetracycline and streptomycin. Several studies have been performed in Iran, showing high frequency of resistance to ciprofloxacin and nalidixic acid for *S. enterica* and *S. infantis* (10, 26). Resistance to these antibiotics has been reported in many studies in other countries (27, 28); however, resistance rate in our study was considerably higher in comparison with other studies. In the Nogrady et al. investigation, resistance to nalidixic acid was reported to be at MIC ≥ 256 µg/L while it was at MIC > 2048 µg/L in the present study (6). Also our findings indicate that 16% of strains were resistant to at least four, and 12% to nine antibiotics. 

In the studied strains, 22 antibiotic resistance patterns were observed and the most common anti-biotypes (34%) were associated with the phenotype of resistance to six antibiotics. In a study by Dahshan et al. on *S. infantis* strains, resistance pattern was reported to be to AMP, CHI, STR, SUL, TE, CEF, FOX and CAZ (28). The reason for increasing resistance among species that can cause food infection is indiscriminate and uncontrolled use of antibiotics for medical and veterinary purposes which causes destruction of sensitive bacteria and selection of resistant strains to several antibiotics. These strains can directly infect human through food consumption and can transfer resistance genes to human endogenous flora (29). Restriction in the use of antibiotics in animals and humans, antibiotics sensitivity testing for the selection of appropriate drug (s), and observing the dose of medications and treatment duration can decrease the number of resistant strains.

Up until now, more than 80 resistance genes have been identified for class I integrons which confer the resistance of the bacteria to different antibiotics. Our findings revealed that 100% of integron-positive strains were resistant to nalidixic acid, tetracycline and streptomycin. But other than that, except for ceftazidime, no significant association was observed between the presence of class I integron gene and antimicrobial resistance. The association of resistance gene and class I integrons in *S. infantis* has been described in several studies (10, 30, 31). In a study by Naghoni et al. conducted on clinical (samples), 96% of integron-positive samples were resistant to tetracycline and 83% to streptomycin (8). In another study by Japanese researchers, resistance to streptomycin and tetracycline in *S. infantis* isolated from chicken was associated with the presence of class I integron (32, 33). 

Different resistance genes have been identified in *S. infantis* (34). These observations indicate the relationship between the size and the source of class I integrons and efflux proteins encoding resistance genes in this bacteria (28). Although transmission of resistance in *Salmonella *infections is complex and not exactly clear, most resistant *Salmonella* infections result from contaminated food of animal origin (6). These resistant bacteria in foods are considered as a threat for human’s health; and therefore, it is recommended that antibiotic resistance genotypes and their association with class I integrons be evaluated in the strains resistance to multiple antibiotics.

In our study, the rate of antibiotic resistance has been high among *S. infantis* strains and a great number of these isolates have been found with multi-antibiotic resistance. Monitoring and surveillance of antimicrobial resistance including integron screening as an indicator of resistance acquisition can be an important strategy to combat antibacterial resistance among these microorganisms.
